# Synthesis and Characterization of Triticale Starch-Based Hydrogel for pH Responsive Controlled Diffusion

**DOI:** 10.1021/acsomega.4c02536

**Published:** 2024-06-21

**Authors:** Karen S. Cruz-Amaya, Diego Hernández-Martínez, Carmen L. Del-Toro-Sánchez, Elizabeth Carvajal-Millan, Karla Martínez-Robinson, Yubia B. DeAnda-Flores, Yaeel I. Cornejo-Ramírez

**Affiliations:** † Departamento de Investigación y Posgrado en Alimentos, Universidad de Sonora, 83000 Hermosillo, Sonora, Mexico; ‡ Departamento de Investigación en Polímeros y Materiales, Universidad de Sonora, 83000 Hermosillo, Sonora, Mexico; § Centro de Investigación en Alimentación y Desarrollo (CIAD, A.C.), 83304 Hermosillo, Sonora, Mexico

## Abstract

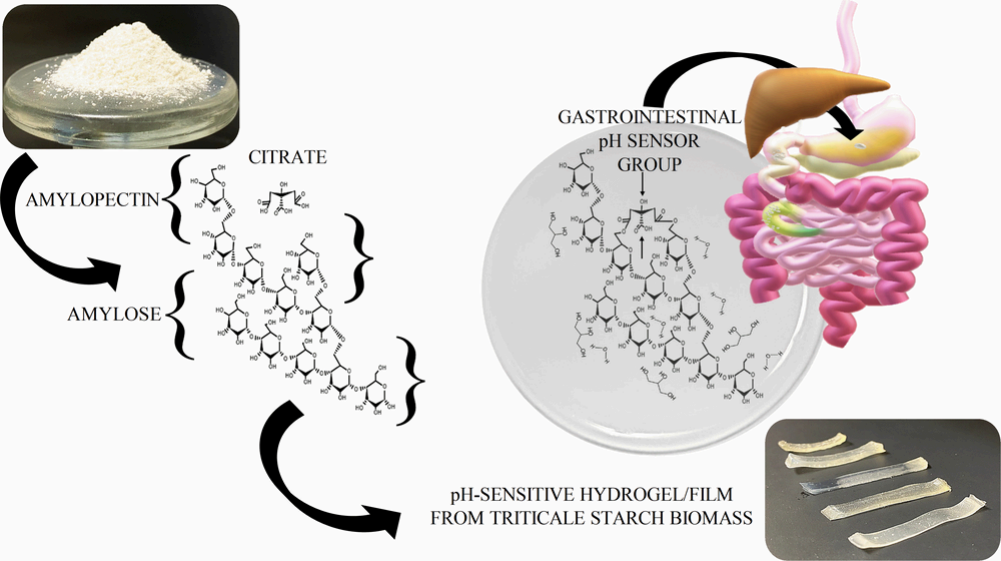

Considering the FAO perspectives for agriculture toward 2030, many natural sources will be no longer profitable for the synthesis of many biomaterials. Triticale (*X Triticosecale* Wittmack) is a cereal crop synthesized to withstand those marginal conditions; however, it is primarily used as fodder worldwide. We reported for the first time the synthesis of a natural anionic hydrogel with gastrointestinal pH stimulus-response as a new alternative of smart material, based on Eronga triticale starch as sustainable biomass, using citrate (p*K*_a_ ∼3.1, 4.7, and 6.4) as cross-linking agent. The scanning electron microscopy and X-ray diffraction exhibited A and B-type starch granules, and semicrystallinity A-type. The presence of the anionic sensing group (COOH) was verified by infrared spectroscopy, the interactions by hydrogen bonds between starch and glycerol and esterification between starch and citric acid were identified by ^1^H NMR spectra, and through thermal analysis hydrogels exhibited four endothermic curves (179–319 °C, ∼0.711–39 kJ/mol *E*_a_). The results showed that the slight addition of glycerol increases the thermal stability, but a higher amount of glycerol decreases the intermolecular forces affecting the thermal stability contrary, the mechanical properties could be benefited. The rheological analyses showed viscoelastic tendency (*G*′ > *G*″) with high stability (Tanδ < 1) in frequency, time, and strain sweeps. Gastrointestinal pH sensitivity (∼2–7.8) was verified (α ≤ 0.01) following Fick’s diffusive parameters, which resulted in a tendency to gradually release BSA with increasing pH ∼3–7 by anomalous and case-II diffusion, showing greater release at pH ∼7.8/3.5 h (80–96%). We aim to expand the biomaterials area focusing on triticale starch due to its limited reported investigations, low-cost, green modification, and its rheological performance as plastic.

## Introduction

Triticale (*X Triticosecale* Wittmack) is an artificial cereal synthesized by crossing the genomes of the diverse varieties of wheat (ABD) with rye (R), inheriting nutrients, the functional properties of wheat and resistance through a diverse marginal environments of low temperatures, high wind conditions, and water stress.^1^ The main worldwide application is as animal fodder, and according to the most recent global statistics from FAOSTAT^2^ the number of cultivated hectares exceeds its production, which makes triticale crops a renewable source. Specialists agree on the high quantity of triticale ears unexploited, where the main biomass of the grain is starch.^3^ Starch is the second most abundant plant reserve polysaccharide in nature, composed of two polymeric chains through glucose monomers and glycosidic bonds: amylose (linear bonds α-D-1,4) and amylopectin (bonds α-D-1,4 and branched α-D-1,6 bonds). The suspension of the starch granules in an aqueous solution exposed to temperatures greater than 60 °C causes the debranching of chains and the consequent retention of water; this gelatinization effect forms reticulated structures with swelling capacity called hydrogels.^4,5^ There is much economic interest in hydrogels due to their wide application (15.33 billion USD forecast in 2022, with a 5% of annual rate toward 2026), and those with stimulus-response to the pH factor are among the most studied in the pharmaceutical area due to their application as oral excipients.^6,7^ Considering that 90% of drugs are absorbed at pH ∼6–7.4, a large number of semisynthetic hydrogels from various natural sources with stimulus-response to intestinal pH have been reported,^8−11^ however, their synthesis involves complex techniques that are not very replicable on a large scale, with corrosive catalyst reagents, slightly toxic and poorly degradable synthetic copolymers, which causes a progressive environmental impact. Starch is a drug excipient recognized by the International Pharmaceutical Excipients Council (IPEC),^12^ with a wide reactivity reported for its modification by green chemistry, such as its cross-linking with citrate (p*K*_a_ ∼3.1, 4.7, and 6.4) by the application of high heat treatment (≥90 °C) allowing the formation of starch-citrate due to the diester bonds (anionic nature).^13^ Furthermore, the natural effect of retrogradation produces the so-called resistant starch, which limits the hydrolysis by α-amylases.^9^ Taking advantage of these functionalities and the high plastic-gelatinization capacity of triticale starch, the synthesis of a natural hydrogel with sensitivity to the gastrointestinal pH factor (pH ∼2, 3, 4, 6 and 7.8) was developed and reported for the first time using starch-citrate cross-linking, where the main objective of this study was to control the higher release of bovine serum albumin (BSA, protein model) at intestinal pH (pH ∼6–7.8) following Fick’s diffusion laws described by Ritger and Peppas.^14^ In theory, we infer that the synthesis of this smart and natural material will present better viscoelastic resistance compared to the previous reported natural hydrogels, with an optimal cost-functionality ratio, as well as a sustainable and progressive scope if we considered that the climate change effects predicted by the FAO through 2030 will limit the production of many plant sources, which will be no longer profitable to produce biomaterials.^15^ Additionally, to the best of our knowledge, there are only a few reported triticale-based biomaterials, including bioplastics and films, and only one previous study of the native hydrogel of triticale starch has been reported.^16−20^ Finally, our effort aims to promote and expand the study of triticale in biomaterials science with sustainable development, introducing this novel application of triticale starch as a first achievement to produce stimuli-responsive hydrogels.

## Materials and Methods

### Materials

Complete hexaploid triticale grains (Eronga variety, AABBRR, Sonora, Mexico) with 62.5% starch contained 23.9% amylose and 76.1% of amylopectin. Fine and granulated anhydride pure food grade citric acid (DGrace, Mexico) was used as a GRAS cross-linking agent. Glycerol ACS (Fermont, Mexico). Grade II purity water.

### Stage I. Extraction, Morphology, and Physicochemical Characterization of Triticale Starch

Starch was obtained using the acid extraction method described by Meredith et al.^21^ The analysis of the size and morphology was performed using the JEOL JSM-5400LV scanning electron microscope (SEM) with 50–2000X of magnitude, applying 15 kV. Functional group detection was performed using Fourier Transform Infrared Spectroscopy equipment (FT-IR, PerkinElmer Spectrum) operated at 20 °C and using a band range between 500 and 4000 cm^–1^. The degree and type of semicrystallinity pattern were analyzed by X-ray diffraction (XRD) using a D8 advanced diffractometer (Bruker model). The detection region was performed at 2θ with a range between 5 and 70° (see eq S1 of the degree of crystallinity in Supporting Information).

### Stage II. Synthesis, Morphology, and Physicochemical and Rheological Characterization of Anionic Hydrogels Based on Triticale Starch

#### Anionic Hydrogels Preparation

The cross-linked starch-citrate was obtained following the reaction mechanism described in Tharanathan.^13^ The hydrogel preparation consisted of a 7% (w/v) concentration of starch suspension in distilled water with a 1:05 starch: citrate ratio (Table S1 in the Supporting Information). The suspension was first sonicated (30 min) with the subsequent addition of glycerol, followed by magnetic stirring (20 min/110 rpm), and placed in a water bath at 90 ± 1 °C. Finally, the hydrogels were cooled for 120 h/5 °C and stored at room temperature.

#### Physicochemical and Rheological Characterization of Anionic Hydrogels

FT-IR spectrophotometer (PerkinElmer Spectrum) was used to detect the functional groups in the samples. It was operated at 20 °C within a band range of 400–4000 cm^–1^. It was operated at 20 °C within a band range of 400–4000 cm^–1^.

Thermogravimetric analysis (TGA) was performed by placing 3–3.5 mg of samples into a Pyris-1 TGA analyzer operated under a controlled N_2_ atmosphere, with a constant heating system (10 °C/min flow) within a temperature range of 25–600 °C. The activation energy (*E*_a_) was calculated following the Coats and Redfern method,^22^ related to the derivative thermogravimetry (DTG) of the TGA graphs. This procedure is based on the Arrhenius equation along with the integration of the derivatives (see eq S2 in Supporting Information) and the application of the property of logarithms for a first-order reaction (*n* = 1):
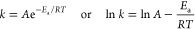
1

where *K* is the reaction rate constant; *A* is the pre-exponential factor (min^–1^); *E*_a_ is the activation energy ; *R* is the universal molar gas constant (8.314 ); and *T* is the absolute temperature (°K).

The rheological characterization was based on the analysis of energy storage (elastic modulus, *G*′) and energy loss-dissipation (viscous modulus, *G*″) using a HR-2 hybrid rheometer (TA Instruments) with a double plate (40 mm diameter) in a parallel position. The analysis was performed through oscillation times of 30 min/25 °C with constant oscillatory tension of 5% and 0.25 Hz; oscillation frequency of 0.01–10 Hz with constant voltage of 5%; and oscillation amplitude of 0.25 Hz with oscillatory voltage of 0.02–20%, respectively.

### Stage III. pH-Sensitivity Assay of Anionic Hydrogels by Apparent Diffusion of Protein

To analyze the diffusion behavior of anionic hydrogels and verify their sensitivity to the gastrointestinal pH factor, a combination of techniques was performed applying the protein release methodology presented in Carvajal et al.,^23^ based on Fick’s second Law described by Ritger and Peppas,^14^ and the Hartree–Lowry assay for protein quantification by a linear absorbance response of UV–vis spectrum, using BSA as release model. The hydrogels (42 ± 0.01 mm diameter and 0.42 ± 0.05 mm thickness) were prepared into 50 mL glass beakers. Subsequently, 500 μL of BSA protein (67 kDa) solution (5000 μg BSA/50 mL 0.05 M citrate-phosphate buffer) was loaded into the hydrogel surface. Protein was allowed to diffuse into the hydrogels with tangential stirring (18 rpm) for 12 h/25–27 °C, and the unloaded solution was rapidly recovered. Then, 6 mL of HCl dissolutions [0.01–0.05 M] and NaHPO_4_ [0.075 M]/KH_2_PO_4_ [0.075 M] buffer at gastrointestinal pH of 2, 3, 4, 6, and 7.8 were added and recovered by decantation after 30 min. It was repeated every 30 min for 3.5 h. The BSA protein released at each period was measured by the Hartree-Lowry assay. The protein release was analyzed by the Fick model (eq 2) to analyze the BSA diffusivity of the hydrogel in each pH system:
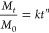
2where *M*_*t*_ is the accumulated mass of protein released at time (t); *M*_0_ is the mass of protein in the hydrogel at time zero;  is the mass of protein released at time (*t*); *t* is the release time; *k* is the release rate constant; *n* is the dissolution exponent characteristic of the system: Case-I, Fickian diffusion, *n* = 0.5. Anomalous diffusion 0.5 < *n* < 1. Case-II, diffusion (relaxation) *n* ≥ 1.

The *M_t_*/*M*_0_ versus the square root of time was plotted for each hydrogel. According to eq 3, if the hydrogel is a plate with a thickness 10 times smaller than the diameter of the container, the diffusion coefficient (*D*_m_) was determined from the linear part of *M*_t_/*M*_0_ (*t*) curves:
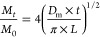
3where *D*_m_ is the apparent diffusion coefficient (); *L* is the initial hydrogel thickness (cm).

### Experimental Design and Statistical Analysis

The experiment was completely randomized design, and data were subjected to a one-way ANOVA method complemented by the Tukey–Kramer HSD test for comparison of means (α ≤ 0.01). The JMP program version 14.0 of SAS (2018) was used for statistics. The Origin Pro-version 9.0 (2012) and SigmaPlot-version 14.0 of SYSTAT (2018) software were used for the graphic design.

## Results and Discussion

### Morphology and Physicochemical Characterization of Triticale Starch

**Figure 1:**
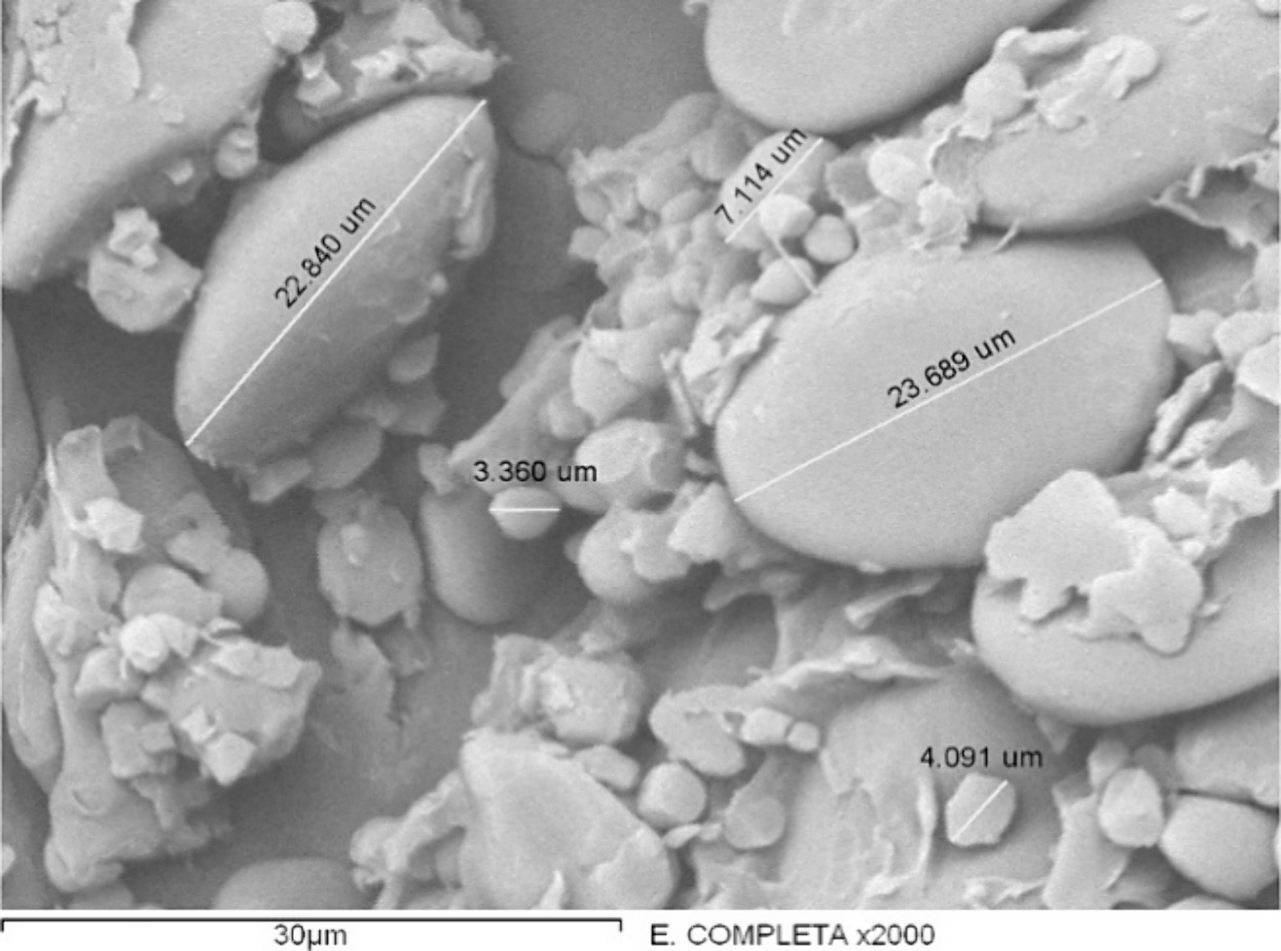
Representative micrographs of Eronga triticale starch granules.

SEM micrographs (Figure 1) allowed to calculate the approximate size distribution frequency of triticale starch granules (Table S2 in Supporting Information), where a right-skewed distribution was observed for the largest A-type granules (10 ≤ 35 μm) with oval-lenticular morphology, followed by a B-type size (∼6 < 10 μm) with spherical morphology. The results were consistent with previous studies.^4,20^

**Figure 2:**
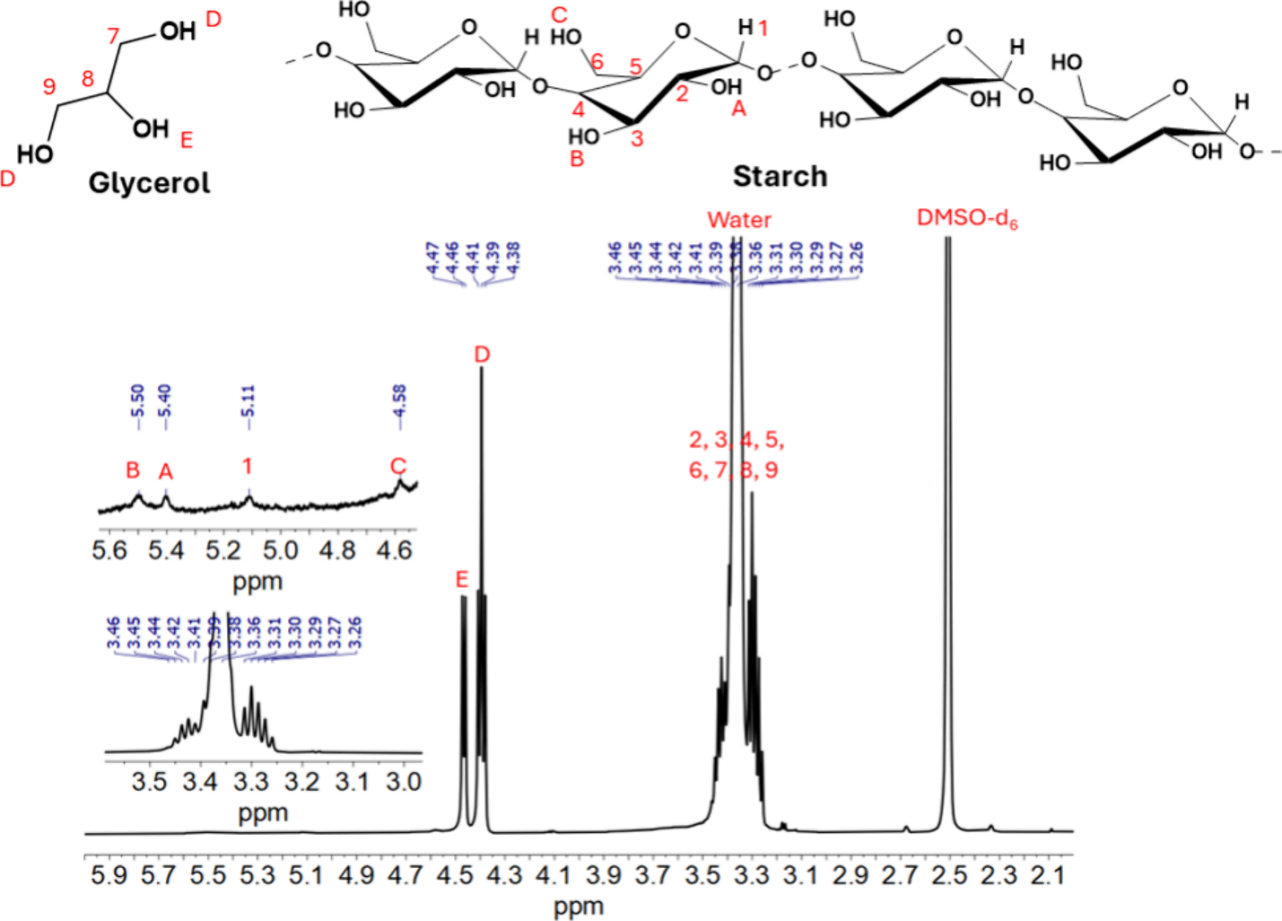
^1^H NMR spectra in DMSO-*d*_6_ for the control sample (starch and glycerol) and the assignment of main peaks.

The ^1^H NMR spectrum of the control sample (with starch and glycerol) is presented in Figure 2. The signals assigned to glycerol are mainly a doublet at 4.47 and 4.46 ppm and a triplet at 4.41, 4.39, and 4.38 ppm corresponding to the OH groups (D and E), also the signals related to the OH of starch at 4.58, 5.40, and 5.50 ppm are observed (labeled in the structure as C, A and B, respectively), as well as the signals of 5.11 and 3.46 ppm corresponding to the α-(1 → 6) bond (labeled in the structure as C, A and B, respectively). 4.58, 5.40, and 5.50 ppm (labeled in the structure as C, A, and B), respectively, as well as the signals at 5.11 and 3.46 ppm corresponding to the α-(1 → 6)-glucosidic linkage, the other signals corresponding to the starch identified by Mondal et al.^24^ are possibly overlapped by the glycerol and water signals between 3.46 and 3.26 ppm.

**Figure 3:**
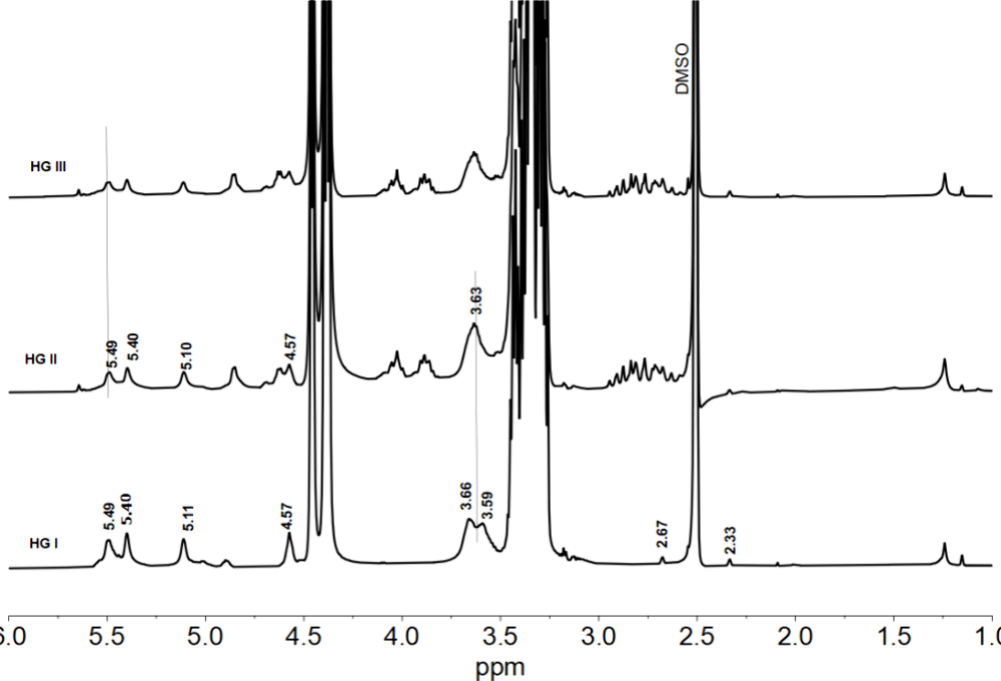
^1^H NMR spectra in DMSO-*d*_6_ for HG-I, HG-II, and HG-III.

Figure 3 shows the ^1^H NMR spectra of materials HG-I, HG-II, and HG-III, all with the same amount of starch and citric acid, and the variation of glycerol (0.50, 0.75, and 1. 00 mL, respectively), as with the control sample, the signals corresponding to glycerol are predominantly observed, since it is more significant proportion than the other components, as well as a slight displacement of its signals at low field, indicative of the possible interaction by hydrogen bonds between starch and glycerol. New signals corresponding to starch OH are also observed at 5.44 and 4.57 ppm for HG-I; 5.48, 5.40, and 4.86 ppm for HG-II and 5.49, 5.40, 4.63, 4.58, and 4.85 ppm for HG-III, suggesting a change in the chemical environment of these OH groups due to esterification between some OH groups of starch and citrate in agreement with the changes reported by Chi et al.^25^ Finally, this proposal of esterification between starch and citric acid is corroborated due to the signals that we can observe at 2.67 and 2.33 ppm of the −CH_2_ of citrate, thus concluding the possible formation of hydrogen bridges between starch and glycerol in addition to the formation of esters between starch and citrate, with a possible structural proposal as reported by Seligra et al.^26^

**Figure 4:**
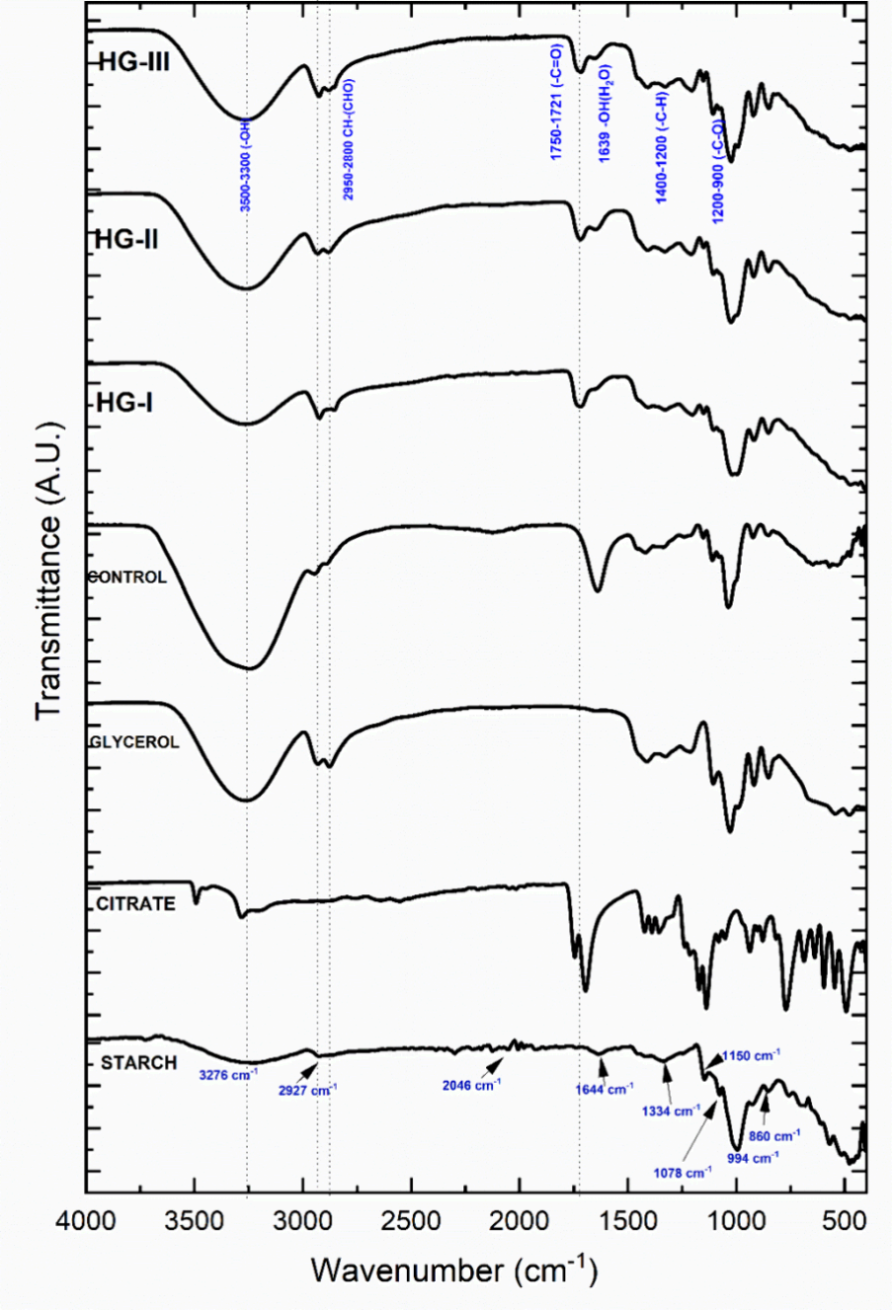
FT-IR for precursors and HG-I, HG-II, and HG-III.

The absorbance bands of bonds and functional groups of the native triticale Eronga starch were obtained by FT-IR spectroscopy (Figure 4); the vibrational spectra of cycloalkane groups (C–C, 900–400 cm^–1^), the α-1,4 glycosidic bonds (C–O–C, 930 cm^–1^), the vibrational stretching of methylene groups (C–H_2_, at 1465 cm^–1^), and a hydroxyl bending by trace moisture in amorphous amylose zones (O–H, 1640–1630 cm^–1^) were identified. Additionally, the presence of the hydroxyl group (O–H) was observed an intense vibrational band at 3400–3300 cm^–1^, and the vibration of the carbon–hydrogen bond (C–H) at 2950–2800 cm^–1^ due to the possible formation of the hydrogen bonds.^27,28^ Despite FT-IR spectroscopy has been previously performed on triticale,^29,30^ to the best of our knowledge, data on the FT-IR spectra of triticale starch (Eronga variety) have not been reported; however, the spectrum was similar to the spectra of different starch sources.^28^

According to Seligra et al.^26^ the amount of OH in a material is determined by FT-IR based on the calculation of the ratio of intensities between I_3272_/I_1149_. The intensity at 3272 cm^–1^ is attributed to the −OH of starch and glycerol, and the intensity at 1149 cm^–1^ is associated with the stretching vibration of C–O and C–O–H. Therefore, the higher the ratio, the higher the OH available. We calculated I_3272/_I_1149_ for starch and glycerol, which resulted in 8.63 for the former and 2.47 for the latter, suggesting a higher availability of −OH in starch. Based on this, starch, which has a higher amount of −OH, reacts with citrate in the esterification process. This is confirmed by the appearance of a new band in samples HGI, II, and III at 1728 cm^–1^ associated with the carbonyl (C=O) formed after esterification, which is indicative of the starch-citrate cross-linking in the hydrogels.

Starch has the following characteristic bands: 3123–3620 cm^–1^ weak H-bonded O–H str., 2927 cm^–1^ −CH_2_– asym. str., 2153–2064 cm^–1^ strong H-bonded O–H str., 1246 – CH_2_OH side chain, 1165 cm^–1^ C–O/C–C str., 1095 cm^–1^ C–O–H vending, 933 cm^–1^ C–O–C vending of α-(1 → 4)-glucosidic, 861 cm^–1^ −CH_2_– def., 767 cm^–1^ C–C str., 713–524 cm^–1^ skeletal mode vibrations of glucopyranose ring,^24^ 1642–1645 cm^–1^ attributed to the hydroxyl group of absorbed water within starch, or this peak might also be linked with O–H stretching vibration groups of glycerol as a plasticizer, as we add glycerol in the HGI, II, and III samples the increase of the band intensity is significant. About a hydrogen bridge interaction, the band at 3276 cm^–1^ shifts at a high frequency of a few units. Another quality is that the band becomes broader at higher and lower wavenumbers and less intense concerning that peak, indicating that OH groups form hydrogen bonds.^31^ Finally, the band between 2929 and 2958 cm^–1^ could be related to the simultaneous prevalence of unconventional C–H···O and conventional O–H···O H-bonds.^32^

**Figure 5:**
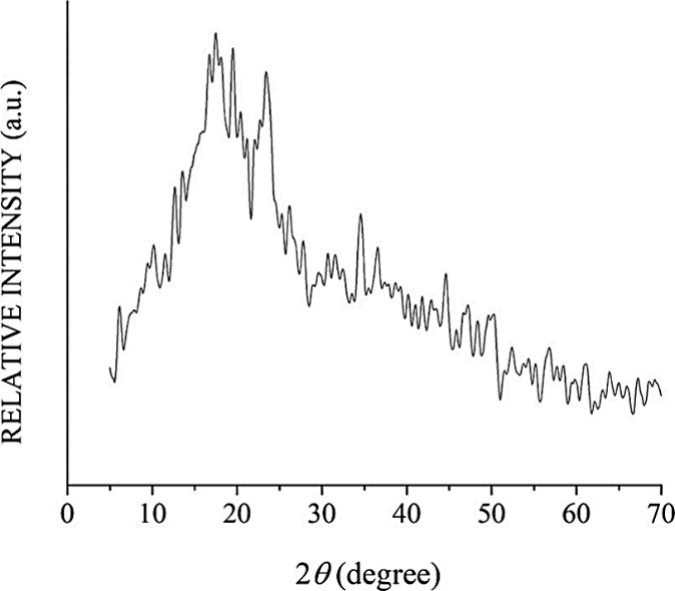
X-ray diffractogram of triticale Eronga starch.

Figure 5 shows the starch diffraction pattern determined by XRD analysis. The highest intensity peaks appeared at 2θ = 12.59°, 16.59°, 17.43°, 18.16°, 19.47°, 23.46°, and 34.54°, which represented 29.26% of the semicrystallinity, similar to the different spectra of triticale varieties previously reported.^20^ These crystal signals are representative of the A-type pattern, which corresponds to a dense structure of parallel amylopectin chains located in a monoclinic plane with less organization. Additionally, this structure has been related to a higher starch reactivity, improving the viscoelastic development in the gel retrogradation process.^4,33^

Finally, the TGA results (Table 1) showed that triticale starch was the most heat-resistant material with an intense endothermic curve in a temperature range of 272–350 °C, with ∼50% of mass degradation (within the maximum peak at 315 °C) and activation energy (*E*_a_) of 126.75 kJ/mol, similar to the TGA/*E*_a_ results from different starch sources.^34^ It has been reported that the first stage of pyrolysis is due to chain depolymerization, followed by ring decomposition and carbonization as the final degradation process. The increase in amylose promotes thermal degradation, so the initial drop in weight was due to catalytic depolymerization of the α-1,4 bond, starting with the lowest molecular weight chains.^34,35^ Although there are recent reports of the thermal characterization of triticale starch by differential scanning calorimeter and hybrid rheometer, those studies only reached gelatinization temperatures (∼60 °C), and by TGA/DTG the thermal decomposition of triticale straw was reported, however, the straw contains polymeric mixtures and diverse biomass of low molecular weight that allowed higher heat resistance (200–400 °C) and *E*_a_ (176.1–213.6 kJ/mol).^20,36,37^

**Table 1:** Degradation Temperatures of the Thermogravimetric Curves and Activation Energies of Hydrogels and Raw Materials^a^

	first curve					second curve					third curve					fourth curve				
material	*T*_o_	*T*_p_	*T*_f_	Δ% *W*^b^	*E*_a_^c^	*T*_o_	*T*_p_	*T*_f_	Δ% *W*^b^	*E*_a_^c^	*T*_o_	*T*_p_	*T*_f_	Δ% *W*^b^	*E*_a_^c^	*T*_o_	*T*_p_	*T*_f_	Δ% *W*^b^	*E*_a_^c^
starch	272	315.0	350	50	126.75															
citrate	157	210.1	252	48	124.89															
glycerol	105	149.8	153	16	34.20	165	200.7	203	79	81.02										
control HG	125	179.8	181	22	38.50	181	229.4	228	44	21.32	228	281.9	300	60	11.91	300	320.9	360	80	39.49
HG-I	125	180.2	181	21	25.87	181	206.4	228	37	33.73	228	278.6	300	62	5.17	300	327.2	360	75	20.86
HG-II	125	178.4	181	25	25.61	181	195.5	228	37	29.92	228	248.7	300	62	8.70	300	324.5	360	80	24.43
HG-III	125	169.7	181	29	33.34	181	196.4	228	51	32.08	228	249.8	300	72	0.71	300	320.8	360	81	15.48

a *T*_o_: onset decomposition temperature (°C). *T*_p_: peak temperature. *T*_f_: final decomposition temperature.

b Δ% *W*: percentage of weight loss in *T*_p_.

c *E*_a_: thermal decomposition activation energy (kJmol^–1^) from *T*_o_–*T*_f_. *R*^2^ range 0.942 ≤ 0.995, Coats and Redfern method.^22^

### Synthesis, Morphology, and Physicochemical and Rheological Characterization of Anionic Hydrogels Based on Triticale Starch

#### Synthesis

**Figure 6:**
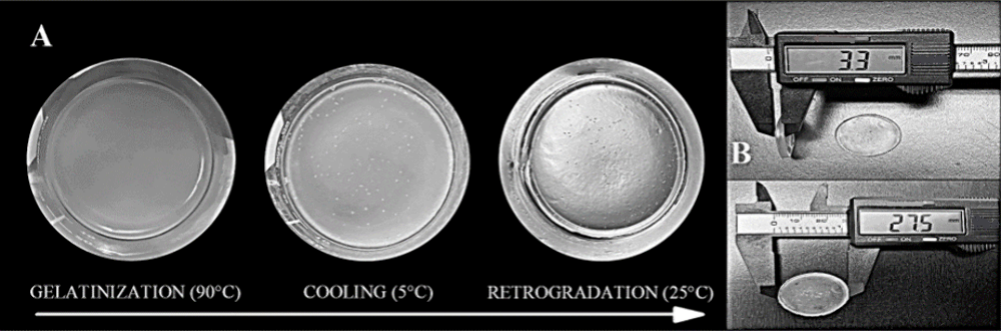
Morphology changes of an anionic hydrogel of triticale starch from gelatinization to the retrogradation process. (A) Recrystallization of starch after cooling. (B) Dimensions (mm) of thickness and diameter after 1 month of storage at 25 °C.

The hydrothermal treatment at 90 °C is a nondestructive process that allowed the gelatinization of the granules and subsequent formation of the starch-citrate. The synthesis was performed based on the effective citrate cross-linked hydrogels reported by Wu et al.^35^ and Duquette et al.^38^ Refrigerated storage (120 h/5 °C) maintained humidity and prevented abrupt rupture of the hydrogel, allowing better stability in the natural retrogradation process (20–25 °C), and showing higher dimensions of thickness (∼6.0 ± 0.52 mm) and diameter (∼39.0 ± 0.16 mm), while xerogels presented reduced dimensions (thickness ∼2.7 ± 0.47 and diameter ∼31.0 ± 3.91 mm) (Figure 6). These results agreed with those reported by Li and Hamaker,^39^ where 120 h of storage for retrogradation of sorghum starch under refrigeration (4°) and freezing (−20 °C) improved the viscoelastic development of the gels. In view of these observations, three experimental HG of similar proportion were selected, labeled as HG-I, HG-II, and HG-III.

### FI-IR, TGA/DTG, and *E*_a_ Analysis

The presence of a new well-defined band due to carbonyl bond vibration (C=O, 1721–1755 cm^–1^) was confirmed by the extrapolation of FT-IR spectra of the hydrogels and the raw material (Figure 4), which is indicative of the starch-citrate cross-linking in anionic hydrogels.^38,40,41^ A higher intensity of vibration was presented in hydrogels within the absorbance range of 500–1220 cm^–1^ due to the bending of C–OH and C–O bonds, related to the decrease of the semicrystalline zone after the gelatinization process.^30^ Furthermore, the control hydrogel showed a higher intensity band at 1639 cm^–1^, representative of the vibration of the water retained in the amorphous zone of native starches. On the other hand, hydroxyl groups (O–H, 3300–3500 cm^–1^) and C–H–O–H bonds (2800–2950 cm^–1^) of lower intensity were presented in the experimental hydrogels, which were attributed to the vibrational stretching caused by the hydroxyl groups in the formation of hydrogen bonds, possibly due to a higher interaction force with water molecules and polymer chains;^42^ for this reason its intensity was lower compared to the spectra of glycerol and the control hydrogel. Moreover, HG-I showed the lowest transmittance due to carboxyl and hydroxyl groups (1750–1721 and 3500–3000 cm^–1^, respectively); in turn, the diffusion results (Table 2) showed that the release behavior was not related to the pH sensitivity. From these behaviors a greater presence of glycerol-citrate esterification was considered, since under the same conditions Halpern et al.^43^ reported this chemical cross-linking at 1724 cm^–1^.

**Table 2:** Cumulative Release and Diffusion Kinetic Parameters of BSA from Hydrogels with Stimulus-Response to the Gastrointestinal pH Factor^a^

material	%^a^	*D*_m_^b^	*n*^c^	*k*^d^	%	*D*_m_	*n*	*k*	%	*D*_m_	*n*	*k*
	**pH 2**				**pH 3**				**pH 4**			
HG-I	92^b^	7.95^b^ ± 0.13	0.96 ± 0.02	0.51 ± 0.11	54^d^	2.61^b^ ± 0.06	0.99 ± 0.01	0.22 ± 0.03	52^d^	2.52^c^ ± 0.40	1.04 ± 0.07	0.13 ± 0.08
HG-II	87^b^	6.56^b^^,^^c^ ± 0.31	0.93 ± 0.00	0.63 ± 0.04	46^e^	1.88^c^ ± 0.04	0.98 ± 0.02	0.20 ± 0.05	70^d^	5.00^b^^,^^c^ ± 0.30	1.00 ± 0.01	0.28 ± 0.02
HG-III	83^d^	6.01^c^ ± 0.02	1.01 ± 0.03	0.26 ± 0.06	40^**e**^	1.19^d^ ± 0.00	0.87 ± 0.00	0.47 ± 0.03	69^e^	4.37^b^ ± 0.11	0.82 ± 0.00	1.41 ± 0.11
	**pH 6**				**pH 7.8**							
HG-I	49^d^	1.91^d^ ± 0.00	0.78 ± 0.00	1.51 ± 0.01	64^c^	3.83^c^ ± 0.09	0.91 ± 0.00	0.55 ± 0.03				
HG-II	72^c^^,^^d^	4.67^c^ ± 0.07	0.77 ± 0.00	2.39 ± 0.16	80^b^^,^^c^	5.46^b^^,^^c^ ± 0.61	0.75 ± 0.01	3.23 ± 0.35				
HG-III	88^c^	5.96^b^ ± 0.15	0.73 ± 0.01	4.09 ± 0.47	96^b^	7.72^b^ ± 0.00	0.73 ± 0.00	4.86 ± 1.12				

a Mean of experimental values ± standard deviation (*n* = 3).

b Total percentage of BSA release (5000 μg/3.5 h) for the difference in pH. Individual rows with different superscript letters indicate significant differences (α ≤ 0.01).

c Apparent diffusion coefficient: *D*_m_ × 10^–9^ (). *R*^2^ 0.95 ≤ 0.99. Individual columns with different superscript letters indicate significant differences (α ≤ 0.01).

d *n*: diffusional coefficient exponent (dimensionless). 0.95 ≤ *R*^2^.

e *k*: release rate constant (s^–1^, first-order reaction). 0.95 ≤ *R*^2^.

**Figure 7:**
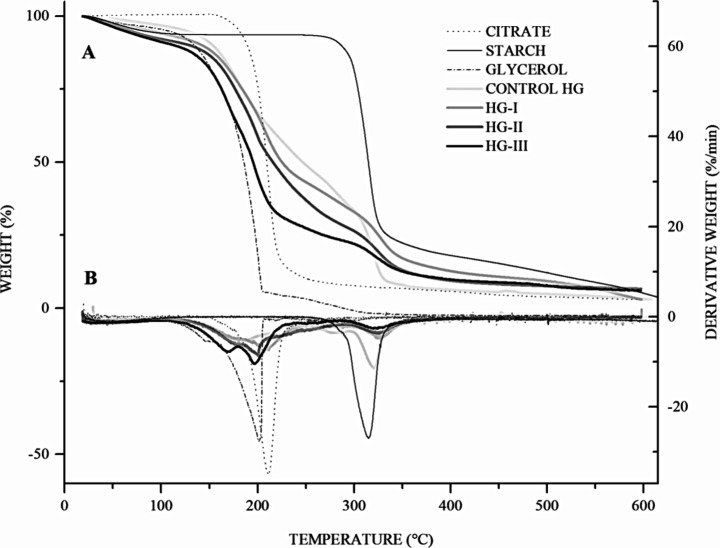
Thermogravimetric kinetics based on thermal decomposition of hydrogels and raw materials. (A) Thermogravimetric curves of weight loss. (B) Derivatives of thermogravimetric curves (DTG) of weight loss as a function of temperature.

The TGA kinetics results presented in Table 1 together with the TGA/DTG (Figure 7) reinforced the previous theory by considering that triticale starch was the most heat-resistant material followed by HG-I, which allowed slightly higher thermal stability (peak temperature *T*_p_ ∼180–329 °C) compared to HG-II and HG-III (*T*_p_ ∼171–326 °C). In turn, a similar *E*_a_ (at the same temperature rate) is necessary for a lower weight drop in HG-I compared to HG-II and HG-III; *E*_a_ can be obtained from the onset and the final decomposition temperature range (*T*_o_ and *T*_f_), while the weight drop can be obtained from the peak temperature *T*_p_ in the DTG (see Figure S1 in Supporting Information for comparative visualization of the *E*_a_). Regarding the three experimental hydrogels, it has been widely reported that starch-citrate cross-links affect thermal stability by modifying the native starch chains,^37^ nonetheless, the DTG of the glycerol-citrate^43^ and starch-citrate-glycerol^42^ cross-links are similar, which makes it difficult to identify them correctly.

Triticale starch-based hydrogels exhibited four characteristic endothermic curves between 179 and 319 °C with *E*_a_ ∼0.711–39.49 kJ/mol. The first weight loss is related to the water evaporation (75–150 °C), followed by the breaking of the acid bond at approximately 200 °C, and consequent pyrolysis of the properties of glycosidic bonds between polymer chains (269–329 °C).^34,35,44^ The degradation kinetic parameters of the present study were slightly similar between the control hydrogel and the experimental hydrogel (Figure 7). At first, the control HG showed slightly higher thermal stability in the range of 25–319 °C. Therefore, it appeared that the lower the native starch, the lower the thermal stability, however, the control HG also showed its higher weight drop in the same temperature range as triticale starch (314–320 °C), allowing the experimental hydrogels to be the most stabilized after that range. Similarly, Meng et al.^45^ reported that hydrogels based on tapioca starch esterified with sodium trimetaphosphate showed a higher thermal stability than native and modified starch after ∼320–330 °C, with a maximum endothermic curve of *T*_p_ ∼260 °C. The study of Wu et al.^35^ showed by differential scanning calorimetry and TGA that citrate cross-linking (5–20%w/w) in starch/chitosan-based films did not present a significant difference in thermal stability (only one peak temperature at *T*_p_ ∼281.1–284.1 °C) compared to non-cross-linked films (*T*_p_ ∼279.2). Those results were lower in thermal stability compared to the triticale starch-based hydrogels (lower weight drop in *T*_p_ ∼228–319 °C), where citrate esterification is an economical and green option, while triticale has less agricultural value. On the other hand, Duquette et al.^38^ reported that citrate-cross-linked starch-based hydrogel had better thermal stability (378 °C, 50% degradation) than native corn starch (306 °C, 50%); however, cross-linking of starch with itaconic acid reduced the thermal stability of the hydrogel. Despite this, their study only reported the DTG thermograms, which does not allow for an analysis of weight loss behavior due to the similar pattern in the decay curves.

The *E*_a_ of the hydrogels was lower compared to starch, possibly due to the previous gelatinization process, where the water and glycerol molecules could influence the separation of the polysaccharide chains, being retained between them during the entire retrogradation process. Supporting the previous assumption, Correa-Pacheco et al.^16^ reported that the addition of glycerol in films containing triticale, starch/potato, and starch/PLA caused a significant increase in thermal conductivity, diffusivity, and effusivity, which could also affect the thermal stability of the hydrogels HG-II and HG-III. Basiak et al.^31^ observed a similar behavior with the glycerol content in the starch highlighting that a higher quantity of plasticizer a lower thermal stability of the material. Jiugao et al.^40^ found that citric acid improves the adhesion between glycerol, water, and starch but could also promote a slight acidolysis of the starch, decreasing the thermal stability of the thermoplastic. Semidegradable thermoplastic films have shown slightly higher thermal resistance (360–460 °C, ∼80% degradation) than many natural hydrogels,^46^ nevertheless, experimental triticale starch-based hydrogels had lower *E*_a_ (0.711–33.73 kJ/mol) for low weight drop over wide temperature ranges (*T*_o_–*T*_f_) but with comparable heat resistance (360–460 °C, ∼84–91% degradation). Finally, the hydrogel samples had the same quantity of citric acid, so the results in this research displayed the possibility that slightly addition of glycerol increases the thermal stability but a higher glycerol content in our hydrogels decreases the intermolecular forces; therefore, the thermal stability in the hydrogels is not benefited.

### SEM Analysis

The hydrogels were analyzed by SEM. A highly irregular and nonporous surface (limited micropores of 2–13 μm) was observed with the presence of concave zones in certain areas. Interestingly, Zhang et al.^47^ observed similar irregular surfaces in seminatural starch hydrogels, where an anionic group and a polyglycol were also added in the synthesis process. Besides, nonporous surfaces were also reported by Duquette et al.^38^ on a natural citrate-cross-linked starch-based hydrogel. Moreover, Yoon et al.^48^ reported that the retrogradation process under refrigeration (4 °C) decreases the porosity, improving the enzymatic resistance to hydrolysis by α-amylases.

### Viscoelastic (*G*′, *G*″) Analysis

**Figure 8:**
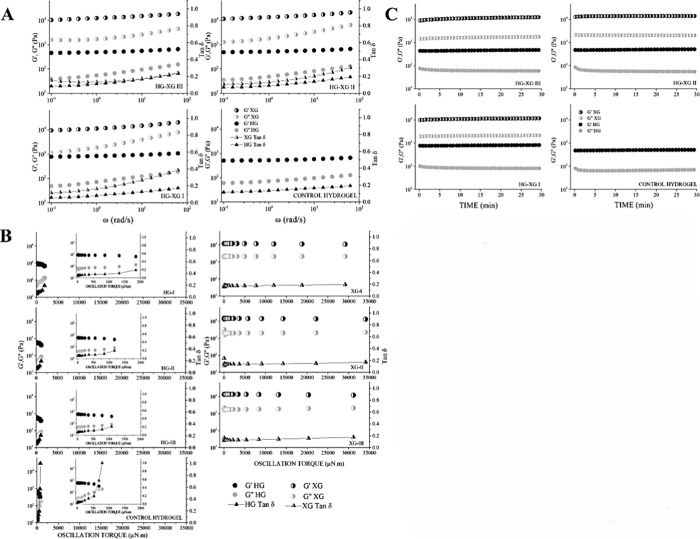
(A) Frequency sweep: viscoelastic moduli of gels and xerogels as a function of angular frequency (ω). (B) Strain sweep: viscoelastic moduli of gels and xerogels as a function of the oscillatory torque force. (C) Time sweep: viscoelastic moduli of gels and xerogels as a function of time with constant stress (5%) and frequency (0.25 Hz).

Figure 8A–C shows the viscoelastic profiles obtained from gels (24 h retrogradation) and xerogels (720 h retrogradation) in the frequency, strain, and time sweep, respectively. In general, the two version of the material exhibited linear *G*′ > *G*″ moduli tendency. A viscoelastic solid-type behavior was observed in the xerogels, related to a better viscoelastic development. However, based on the Tan δ < 1 tendency, stability prevailed in both versions of the material,^44^ except for the control hydrogel, which showed the weakest viscoelastic strength. Additionally, we observed a characteristic glycerol effect depending on the version of the material (HG/XG-I 0.5 mL, HG/XG-II 0.75 mL, and HG/XG-III 1 mL of glycerol). For all studied sweeps in the hydrogels, the less glycerol, the higher viscoelastic force, while for the xerogels, the less glycerol, the lower viscoelastic force (except in the xerogel deformation sweep, where the proportions must be optimal, since an excess or less amount of glycerol can produce slightly undesirable resistance values).

In the frequency sweep, it can be easily observed that the addition of glycerol reduced the viscoelastic modulus in hydrogels (Figure 8A): HG-I (*G*′ ∼781–1078; *G*″ ∼48–188 Pa), HG-II (*G*′ ∼493–675; *G*″ ∼35–131 Pa), and HG-III (*G*′ ∼455–663; *G*″ ∼39–156 Pa). Conversely, the higher amount of glycerol improved the elastic resistance in the version of the material with greater retrogradation: XG-I (*G*′ ∼9244–20,448; *G*″ ∼1054–7936 Pa), XG-II (*G*′ ∼11,400–21,459; *G*″ ∼1273–6566 Pa), and XG-III (*G*′ ∼9972–18,689; *G*″ ∼455–663 Pa). Furthermore, XG-I presented the highest tendency to destabilization due to the highest value of Tan δ ∼0.388 at the highest frequency. The viscoelastic development of both versions and even the control HG (*G*′ ∼508–666 Pa and *G*″ ∼59–128 Pa) was superior to the native triticale hydrogels of the Eronga variety-hexaploid (without glycerol) (*G*′ ∼280–501 Pa and *G*″ ∼16–46 Pa) previously reported.^20^ However, results were inferior to those reported by Seidel et al.^49^ on starch hydrogels copolymerized with carboxymethylcellulose cross-linked with citrate, succinic acid, and tartaric acid, and similar to those cross-linked with glutaric, adipic, and malonic acid; however, all these cross-linked hydrogels received a retrogradation treatment (140 °C) prior to the addition of water, a process that improved their development.

Analogously, in the deformation sweep the xerogels exhibited a higher resistance at a wide deformation force with lower Tan δ (XG-I 29,145 μN m/*G*′_max_ ∼11,209 Pa/Tanδ = 0.196 < XG-III 31,105 μN m/*G*′_max_ ∼11,941 Pa/Tanδ = 0.176 < XG-II 34,049 μN m/*G*′_max_ ∼13,091 Pa/Tanδ = 0.172) compared to hydrogels (HG-I 1855 μN m/*G*′_max_ ∼672 Pa/Tanδ = 0.197 > HG-II 1163 μN m/*G*′_max_ ∼423 Pa/Tanδ = 0.195 > HG-III 1078 μN.m/*G*′_max_ ∼390 Pa/Tanδ = 0.209). Nevertheless, in the deformation sweep of xerogels, a minimal incorrect proportion of glycerol can produce undesired values, as could be observed by comparing XG-III with XG-II. It is possible that free glycerol molecules and the water retention allow higher mechanical destabilization in the HG version, causing less damping; this would explain the antagonistic effect of glycerol, providing viscosity and energy dissipation in gels but unexpectedly allowing a better rearrangement and cohesion between the XG polymeric chains due to the erosion and dehydration process, improving its resistance due to the formation of hydrogen bonds. The aforementioned assumption is based on the study by Smits et al.^50^ who reported a similar effect of glycerol and ethylene glycol in dehydrated and retrograded starch, improving the mobility and rearrangement of the chains, where heating allowed a less arrangement of the plasticizers compared to the interaction at room temperature (4–8 days/20–27 °C) since a greater interaction between the free glycerol molecules and the amylose and amylopectin chains is favored by the formation of hydrogen bonds, which reduce viscosity and improve resistance to shearing and deformation forces. Besides, the bilateral effect of glycerol has already been reported, where its addition increases strength in certain materials and provides weak viscoelastic strength in others.^51,52^ In general, a linear behavior of the moduli *G*′ and *G*″ prevailed in both versions of the material. On the other hand, there can also be observed an instability trend due to an inclination that leads to the crossing of the moduli (*G*″/*G*′=1 or Tan δ = 1) for the control HG (Figure 8B).

The stability at the time sweep was confirmed by applying a tension of 5% with 0.25 Hz for 30 min, where no drastic changes in the structure of the materials were observed (Figure 6C). Triticale starch-based hydrogels were generally more resistant and stable over time (HG-I *G*′_max_ ∼825 Pa, HG-II *G*′_max_ ∼514 Pa, and HG-III *G*′_max_ ∼478 Pa) compared with other natural polymers such as starch-gelatin (*G*′_max_ ∼600 Pa), methylcellulose (*G*′_max_ ∼200 Pa), fibrin (*G*′_max_ ∼200 Pa), collagen (*G*′_max_ ∼100 Pa), and matrigel (*G*′_max_ ∼90 Pa).^53,54^

Glycerol and citric acid are useful to obtain natural films and hydrogels based on cross-linked starch. These substrates can help prevent starch retrogradation, are reliable for human health and the environment compared to harsh chemicals, and allow the design of modified starch for the pharmaceutical, packaging, or food industries. Citric acid increases thermal stability and inhibits starch retrogradation due to the strong hydrogen bond between starch and citric acid. Additionally, citric acid can help reduce glycerol migration, maintain a high swelling, and promote better mechanical behavior.^40,55,56^ These findings agree with the results of this research; thermal stability and mechanical properties were higher in the sample hydrogels than in the control hydrogels (without citric acid). Thus, the greater the amount of glycerol, the greater the mechanical forces increase; on the contrary, the thermal stability decreases due to the decrease in intermolecular forces.

### pH-Sensitivity Assay of Anionic Hydrogels by Apparent Diffusion of Protein

**Figure 9:**
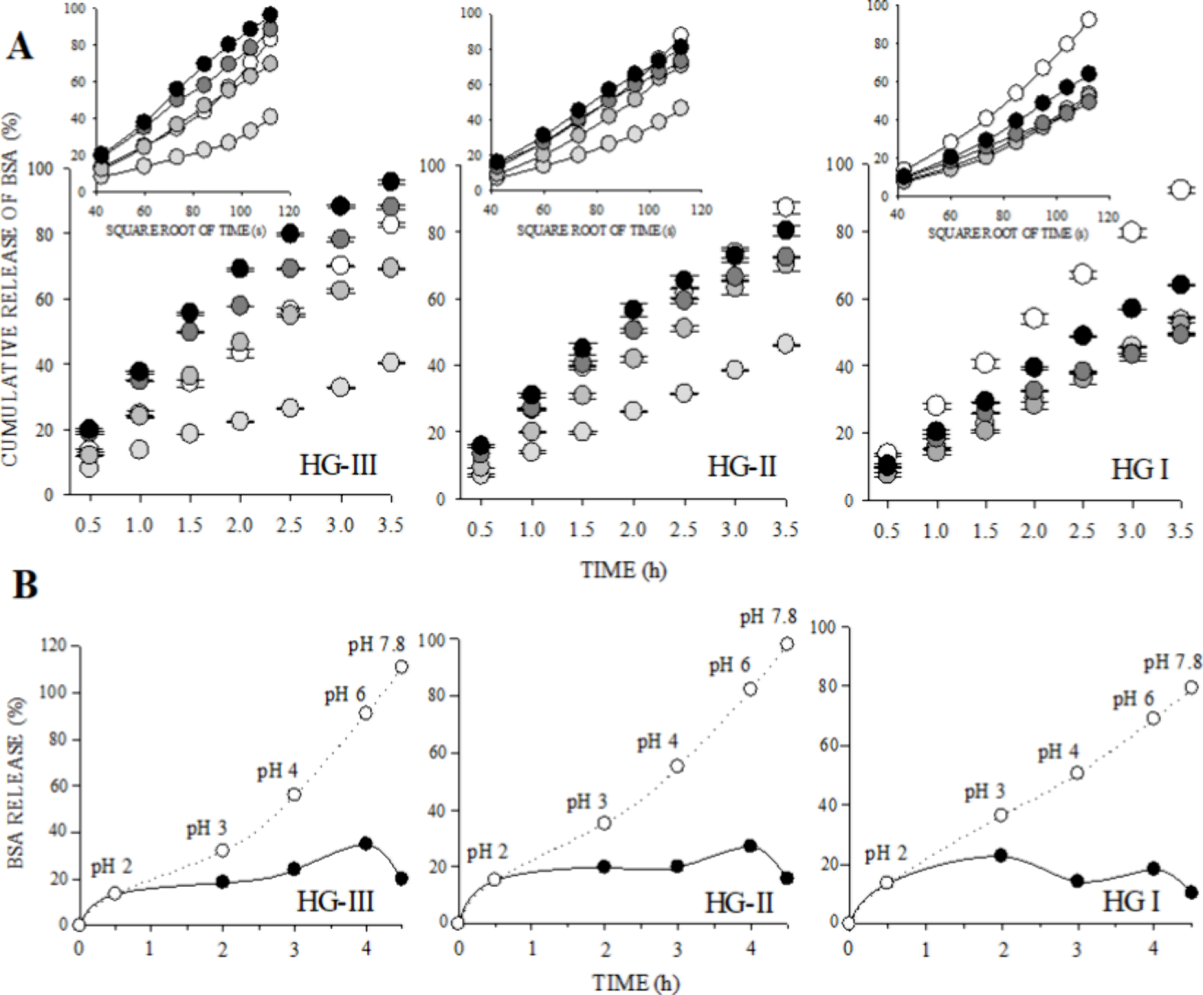
(A) Cumulative release behaviors of BSA from hydrogels in different pH solutions; 2 (open circle), 3 (light-gray shaded circle), 4 (gray shaded circle), 6 (dark-gray shaded circle), and 7.8 (black solid circle). (B) BSA diffusion of hydrogels in a simulated gastrointestinal system; cumulative (open circle) and gradual (black solid circle) release.

Table 2 shows the results of total BSA release, apparent diffusion coefficient (*D*_m_), exponential diffusion coefficient (*n*), and release rate constant (*k*) of BSA from anionic hydrogels in simulated gastrointestinal pH solutions. The diffusion analysis confirmed with significant differences (α ≤ 0.01) the sensitivity to the pH factor of HG-II and HG-III since they exhibited a gradual tendency of BSA released together with the *D*_m_ coefficient with increasing pH ∼3–7.8, achieving a higher release at pH ∼7.8/3.5 h (80–96%) (release behavior of the individual pH medium can be seen in Figure 9A). On the other hand, HG-I did not present a significant difference between the total release and the increase in pH, and inversely, a lower percentage of release was observed at pH ∼7.8/3.5 h (64%); for this reason, the possible cross-linking between starch-citrate was rejected for this hydrogel, and our supposition of the glycerol-citrate cross-linking previously explained and supported by FT-IR, TGA, and rheological characterizations was strengthened. Furthermore, reinforcing the previous analyses, we found a positive and significant Pearson correlation (0.01 ≤ α ≤ 0.05, statistical analysis not attached) between the gradual release of the pH-sensitive hydrogels (HG-II and HG-III) and the increase in pH, while the nonsensitive hydrogel (HG-I) exhibited a negative and nonsignificant correlation. However, we observed that the acidity level of HCl [0.01 M] pH ∼2/3.5 h caused the highest degradation (83–92% release).

Based on the kinetic analysis, the sensitivity behavior of the HG-II and HG-III was reaffirmed since the kinetic parameters in the simulated gastric media tend to *n* ∼1 (pH ∼2–4, 0.82 ≤ *n* ≤ 1.0), indicative of a controlled release,^57^ while its speed kinetics *k* was lower (0.02 ≤ *k* ≤ 1.41 s^–1^). Conversely, the kinetic values of the nonsensitive hydrogel approached *n* ∼1 with increasing pH, showing varied *k* values. Generally, a greater tendency for anomalous diffusion behavior (non-Fickian, 0.73 ≤ *n* ≤ 0.99) was displayed within the gastrointestinal pH range, which is a combination of Fickian diffusion (case-I, *n* = 0.5) and chain relaxation.^57,58^ In experimental studies, case-I has been correlated to the diffusion of the active compound due to the swelling of the polymer network controlled by the osmotic diffusion of particles throughout channels and pores.^9,57,58^ However, considering the null or minimal porosity presented in the SEM micrographs, our conjecture leads us to reject a controlled release mechanism by osmosis (case-I). On the other hand, case-II diffusion (*n* = 1.0) was also observed. In diffusion studies, it is expected to achieve a case-II diffusion (zero-order) because it has been correlated to a greater control of the release by chain relaxation and being independent of the time in any geometric form, although it is difficult to achieve since it allows a constant release.^14,46^

On the other hand, since the diffusion kinetic *k* has s^–1^ units, a short release time leads to a higher *k* value;^59^ this can be observed for the sensitive hydrogels in the simulated intestinal pH medium. The statistical parameter of 0.95 ≤ *R*^2^ confirmed the correct adjustment of the diffusion behavior by the Ritger and Peppas method,^14^ where the linear tendency of the cumulative release over time and the square root of time (used to calculate *D*_m_) are shown in Figure 9A. This is also supported by the kinetics reported by Elvira et al.^46^ and Kalendova et al.^11^ wherein the behavior of natural starch-based hydrogels does not follow a linear tendency of release and swelling over a range of pH ∼3–9.

Figure 9B follows a mechanism of the in vitro release of BSA through gastrointestinal pH changes (pH ∼2–7.8). For the gastrointestinal pH simulation, a gastric residence time of 3 h (gastric emptying of liquid *T*_1/2_ = 80.5 min) was considered, with rapid increase in pH from ingestion (pH = 1.2–3) to pylorus (pH = 4–6.5). Additionally, a subsequent transit time of 11 h for the small intestine, considering 3 units of pH increase from the pylorus to ileocecal valve (pH = 8.7).^60,61^ Following these conditions, by Figure 9B, it is possible to observe that hydrogels could reach an optimal and limited release of 54.7%^a^(HG-III), 42.8%^b^(HG-II), and 28.7%^c^(HG-I) within pH ∼6–7.8 (superscript letters after the following percentages were significantly different at an alpha level of α ≤ 0.01), that represents the release from pylorus to subsequent transit time of 1.5 h, which could also be considered the pH of the small intestine. Despite the characteristics of resistant starch, the enzymatic activity of pepsin and pancreatin could reduce the release of BSA. It is worth mentioning that subsequent heat treatment at 80 °C after starch retrogradation, as well as nonporous surfaces, enhance the resistance effect against α-amylases, which are additionally inactive at pH ∼3.8.^9,62^

A wide variety of studies on the synthesis of semisynthetic polymers with stimulus-response to the pH factor have been reported; their results point to an optimal control release at pH ∼1.2–2 (<10–60%) and pH ∼7.4 (60–92.6%), within 8–24 h through in vitro gastrointestinal conditions.^8−11,46^ However, most of the syntheses of these semisynthetic materials reported the use of cross-linking agents and copolymers with a certain degree of toxicity (methacrylate acid, 2-hydroxyethyl methacrylate, acrylic, N,*N*-methylene-bis-acrylamide, tetraethyl orthosilicate, glutaraldehyde, 2-acrylamido-2-methylpropanesulfonic acid, acryloyl chloride, and epichlorohydrin), as well as unconventional and corrosive initiators such as ammonium persulfate, with low reproducibility on a large scale. In comparison, the release behavior of triticale hydrogels was not optimal due to the short release time of 3.5 h, but it was more sensitive at pH ∼7.8 (80–96%/3.5 h) (Table 2 and Figure 9A).

This is the first report of a natural pH-sensitive hydrogel based on triticale starch, nevertheless, the synthesis of two potential natural materials (starch/ethylcellulose and pectin/carboxymethylcellulose) was previously found with slightly complex techniques and nontoxic reagents with resistance to enzymatic hydrolysis and an optimal control release (pH ∼1.2/≤5% and pH ∼7.4/25–70%, for 8–12 h).^57,62^ We assume that by following these prior techniques, it will be possible to develop an optimal natural hydrogel that will be comparable in sustained release with cost-effective production.

## Conclusions

In this study, Eronga (hexaploid) triticale starch exhibited A-type characteristics (size and crystallinity) that allowed the synthesis of a pH responsive hydrogel based on triticale starch through its cross-linking with citrate, showing better stability than many natural hydrogels previously reported. The pH-sensitive hydrogels (HG-II and HG-III) exhibited anomalous and case-II diffusion releases through gastrointestinal pH, mainly influenced by relaxation of the polymeric chains, reaching total cumulative BSA releases of 72–88 and 80–96% through an intestinal pH solution of pH ∼6 and pH ∼7.8 per 3.5 h, respectively. Furthermore, by 4.5 h in vitro gastrointestinal simulation (changing the pH ∼2–7.8), sensitive hydrogels reached a total release of 97.7–100%, where 42.8–54.7% represented the optimal release at intestinal pH ∼6–7.8/1.5 h. Diffusional analyses justified its potential use as a smart release system; thus, a new application of triticale starch was successfully introduced.

## Supporting Information Available

Supplementary experimental details, methods, and equations for crystallinity by XRD and *E*_a_ by TGA/DTG; size distribution of triticale starch granules; optimal factors for the synthesis of triticale starch-based anionic hydrogels; thermogram of TGA/DTG curves; and linear regression plots of *E*_a_ (PDF)

## Abbreviations

HG: hydrogel
XG: xerogel
BSA: bovine serum albumin
FT-IR: Fourier transform infrared spectroscopy
XRD: X-ray diffraction
SEM: scanning electron microscopy
TGA: thermogravimetric analysis
DTG: derivative thermogravimetric
*E*_a_: activation energy
*D*_m_: apparent diffusion coefficient
*n*: diffusional coefficient exponent
*k*: release rate constant
