# Supplementary material for: Synthesis and Characterization of Triticale Starch-Based Hydrogel for pH Responsive Controlled Diffusion
Source: ACS Omega. 2024 Jun 21;9(26):28564–76. doi: 10.1021/acsomega.4c02536 (PMC11223258; doi:10.1021/acsomega.4c02536)
Supplement: Supplementary file 1 — ao4c02536_si_001.pdf [file ao4c02536_si_001.pdf]

SUPPORTING INFORMATION

Synthesis and Characterization of Triticale Starch-Based Hydrogel for pH Responsive Controlled Diffusion

Karen S. Cruz-Amaya[a], Diego* Hernández-Martínez[b*], Carmen L. Del-Toro-Sánchez[a], Elizabeth Carvajal-Millan[c], Karla Martínez-Robinson[c], Yubia B. DeAnda-Flores[c], Yaeel I. Cornejo-Ramírez[a*].*

[a]Departamento de Investigación y Posgrado en Alimentos, Universidad de Sonora, Hermosillo, C.P. 83000. Sonora, México.

[b]Departamento de Investigación en Polímeros y Materiales, Universidad de Sonora, Hermosillo, C.P. 83000. Sonora, México.

[c]Centro de Investigación en Alimentación y Desarrollo (CIAD, A.C.), Hermosillo, C.P. 83304. Sonora, México.

*Email: yaeel.cornejo@unison.mx
*Email: diego.hernandez@unison.mx
*Email: karenscruz@arizona.edu

Pag. Supporting Information Content

S2…… Equation (Eq. S1)

S3…… Tables S1

S3……. Lyophilization pre-treatment description

S3..…… Equation (Eq. S2)

S4……. Table S2

S5……. Figures (Figure S1)

SUPPLEMENTARY EXPERIMENTAL DETAILS, METHODS, AND EQUATIONS

MATERIALS AND METHODS

Materials

Stage I. Extraction, Morphology and Physicochemical Characterization of Triticale Starch

To calculate the percentage of crystallinity by the XRD pattern of starch granules, the formula based on Nara, Mori & Komiya (1978) (Frost et al., 2009; Dome et al., 2020) was followed, using the software Origin Pro-version 9.0 (2012).

Equation S1. $$\% \text{ Crystallinity} = \left(\frac{Ac}{(Ac+Aa)}\right)(100)$$

Ac: Crystallinity area obtained by diffractogram.

Aa: Amorphous area in the diffractogram.

Stage II. Synthesis, Morphology and Physicochemical and Rheological Characterization of Anionic Hydrogels Based on Triticale Starch

General method of preparation

Table S1. Optimal factors for the synthesis of triticale starch-based anionic hydrogels.

Label	Proportion				Heat Treatment (water bath)		Magnetic Stirring	Sonication
	Starch (g)	Citrate (g)	Glycerol (mL)	Water (mL)	Time (min)	Temperature (°C)	Time (min)	
Control-HG	0.35	-	0.50	4.50	9	90°C±1	20	30
HG-I	0.35	0.175	0.50	4.50				
HG-II	0.35	0.175	0.75	4.25				
HG-III	0.35	0.175	1.00	4.00				

Starch proportion: 7% $^{w}/_{v}$
Starch-Citrate ratio: 1:05

Morphological, physicochemical, and rheological characterization

Lyophilization pre-treatment for SEM analysis. A lyophilization treatment was applied to ~0.1 mm^3 hydrogel cubes by instant cold dehydration (-125ºC) immersing the samples in liquid nitrogen (777-cryo, Brymill cryogenic systems) for 1 min, and subsequently introduced to the lyophilizer equipment (4.5L/-50°C capacity, Labconco brand) for 24 h.

Equation S2: Activation energy (Ea) of the thermal degradation reaction by TGA/DTG

Applying derivatives to study the change in the reaction, we obtain:

$$\frac{d\alpha}{dt} = \frac{\beta\left(\frac{d\alpha}{dt}\right)}{dT} = k(T)\cdot f(\alpha) = Ae^{\frac{-Ea}{RT}}\cdot f(\alpha)$$

Where $\frac{d\alpha}{dt}$ is the derivation of the rate of weight Change (α) in the time (t). Considering that the conversion ratio of the weight change is α=$\frac{w_0+w}{w_0+w_f}$, where w_0 is the initial recorded sample weight, w the current weight at any point in the thermogram, w_f final weight after pyrolysis. β represents the heating flux which is constant in the present study ($\frac{dT}{dt} = 10°C\cdot min^{-1}$), and considering the function $f(\alpha)$=1-α.

The integration gives the new function g(a):

$$g(\alpha) = \int_0^{\alpha} \frac{d\alpha}{f(\alpha)} = \frac{A}{\beta}\int_0^{T} e^{\frac{-Ea}{RT}} dT$$

And applying the logarithmic properties:

$$ln\left(-\frac{(ln(1-\alpha))}{T^2}\right) = ln\left(\frac{AE_a}{\beta R}\right) - \frac{E_a}{RT}$$

Where the form of graphic representation based on the TGA/DTG will be:

$$ln\left(-\frac{(ln(1-\alpha))}{T^2}\right);\ Y\ axis$$

$$\frac{1}{T};\ X\ axis$$

From the linear regression it is possible to calculate the *E*a through the intercept and the slope which represent lnA and $\frac{-Ea}{R}$, respectively.

RESULTS

Synthesis, Morphology and Physicochemical and Rheological Characterization of Anionic

Table S2. Size distribution of triticale starch granules

Scale	Lenght (µm)				Mean Surface Area (µm²)
	f_{min}	f_{mean}	f_{max}	Mean	
300 µm	4.0-11.5	11.5-20.6	20.6-35.7	16.2± 6.1	243.7 ± 160.3
100 µm	2.7-4.7	4.7-17.0	17.0-30.7	10.9 ± 7.1	126.4 ± 134.0

$N_{Scale:300\ \mu m}$=1,037 granules (888 A-type, 135 B-type, 14 C-type)
$N_{Scale:100\ \mu m}$=360 granules (166 A-type, 75 B-type, 120 C-type)

Synthesis, Morphology, Physicochemical and Rheological Characterization of Anionic Hydrogels Based on Triticale Starch

FI-IR, TGA/DTG and *E*a analysis

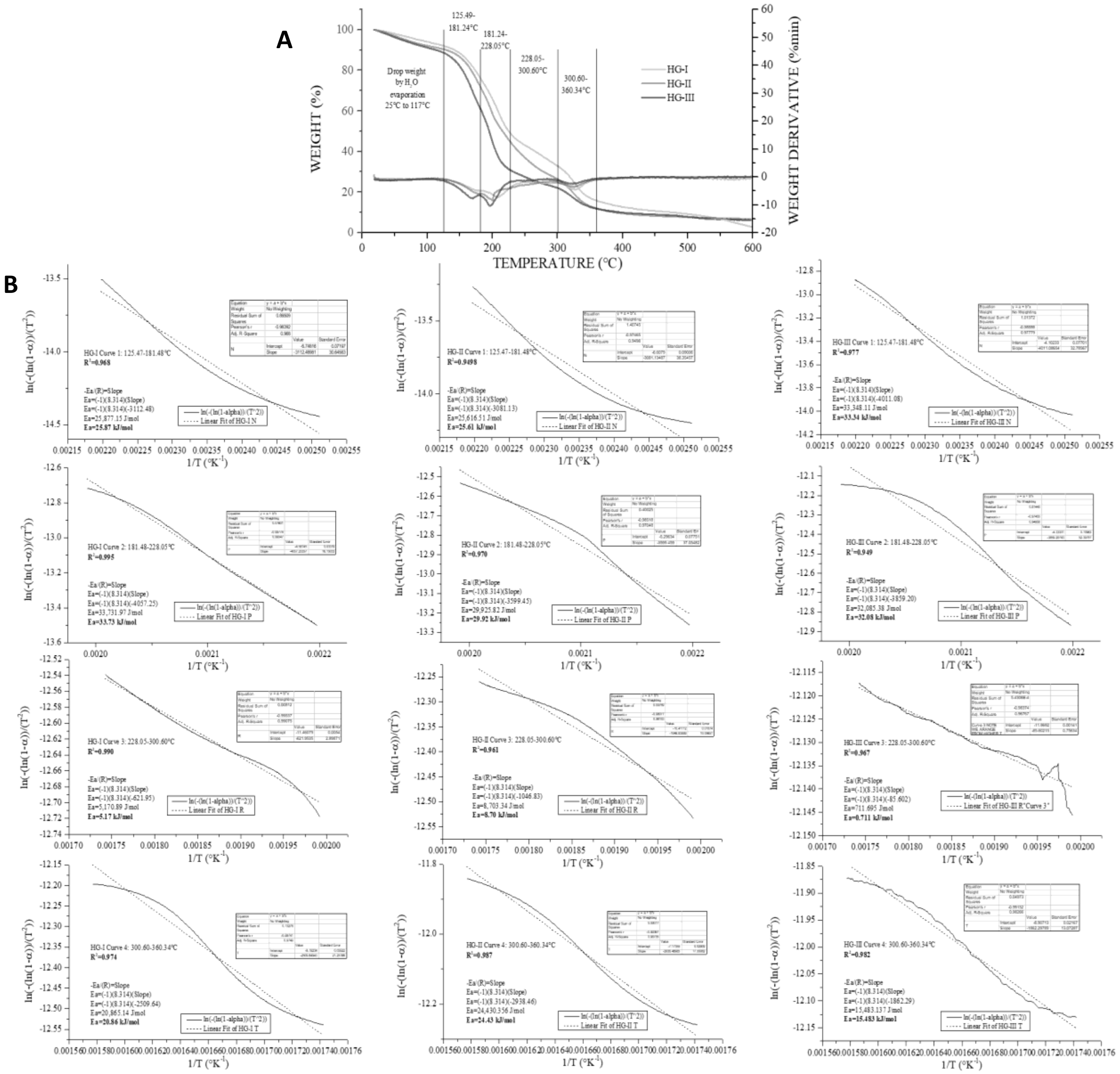

Figure S1. ^{A}Thermogravimetric kinetics and representative drop weight by T_p to calculate the *E*a of the experimental hydrogels. ^{B}Column of graphics ($\frac{\ln(-(\ln(1-\alpha)))}{T^2}$ vs $\frac{1}{T}$) from the 1st to the 4th endothermic curves obtained by the TGA/DTGs kinetics.
